# That Escalated Quickly—Planning to Ignore RPE Can Backfire

**DOI:** 10.3389/fphys.2017.00736

**Published:** 2017-09-22

**Authors:** Maik Bieleke, Wanja Wolff

**Affiliations:** ^1^Social Psychology & Motivation, Department of Psychology, University of Konstanz Konstanz, Germany; ^2^Sport Psychology, Department of Sport Science, University of Konstanz Konstanz, Germany

**Keywords:** Ratings of Perceived Exertion (RPE), endurance performance, psychobiological model, implementation intentions, self-regulation, borg scales, mixed-effects models

## Abstract

Ratings of perceived exertion (RPE) are routinely assessed in exercise science and RPE is substantially associated with physiological criterion measures. According to the psychobiological model of endurance, RPE is a central limiting factor in performance. While RPE is known to be affected by psychological manipulations, it remains to be examined whether RPE can be self-regulated during static muscular endurance exercises to enhance performance. In this experiment, we investigate the effectiveness of the widely used and recommended self-regulation strategy of if-then planning (i.e., implementation intentions) in down-regulating RPE and improving performance in a static muscular endurance task. 62 female students (age: *M* = 23.7 years, *SD* = 4.0) were randomly assigned to an implementation intention or a control condition and performed a static muscular endurance task. They held two intertwined rings as long as possible while avoiding contacts between the rings. In the implementation intention condition, participants had an if-then plan: “If the task becomes too strenuous for me, then I ignore the strain and tell myself: Keep going!” Every 25 ± 10 s participants reported their RPE along with their perceived pain. Endurance performance was measured as time to failure, along with contact errors as a measure of performance quality. No differences emerged between implementation intention and control participants regarding time to failure and performance quality. However, mixed-effects model analyses revealed a significant Time-to-Failure × Condition interaction for RPE. Compared to the control condition, participants in the implementation intention condition reported substantially greater increases in RPE during the second half of the task and reached higher total values of RPE before task termination. A similar but weaker pattern evinced for perceived pain. Our results demonstrate that RPE during an endurance task can be self-regulated with if-then plans. This finding is particularly important given how frequently RPE is used in exercise science as a correlate of physiological processes that ultimately limit performance. Unexpectedly, participants with implementation intentions reported higher RPE than control participants. This suggests that strategies to self-regulate RPE might have ironic effects that hamper performance, maybe by increasing attention to RPE. This implication is important for exercise physiologists, athletes and coaches.

## Introduction

Physiologists have long sought to find the primary determinant of exercise termination (Marcora and Staiano, 2010). This research has mainly focused on “cardiovascular, respiratory, metabolic, and neuromuscular mechanisms of muscle fatigue” (Marcora and Staiano, 2010, p. 768). Offering a more psychological perspective, Swedish psychologist Gunnar Borg published a paper where he suggested that perceived exertion is “the single best indicator of the degree of physical strain” (Borg, 1982, p. 377). To measure the rating of perceived exertion (RPE), he published the now famous *Borg scales* (Borg, 1982, 1998). In short, Borg scales are a set of psychophysical self-report scales with different psychometric properties that can be used to assess different aspects of perceived exertion (Borg, 1970, 1982).

A substantial body of evidence underlines the claim that RPE is an indicator for physical strain (e.g., Chen et al., 2002; Scherr et al., 2013; Moscatelli et al., 2015). In their meta-analysis, Chen et al. analyzed the association of RPE with different physiological criterion measures of physical exertion. Reported validity coefficients for the relationship of RPE with heart rate, blood lactate, %VO_2_max, or respiration rate ranged between 0.57 and 0.72. Thus, self-reported RPE does correspond to ones' internal physiological state. Some inconsistencies in the RPE literature notwithstanding (Noble and Robertson, 1996), the importance of RPE as an indicator of physical strain is now generally accepted (Pageaux, 2014).

Given the validity of RPE as an indicator of strain, it has been proposed (e.g., Scherr et al., 2013) and utilized (Foster et al., 1996) as a cost effective tool for monitoring training load. For example, Impellizzeri et al. (2004) showed that RPE per training session was a valid indicator of training load in a sample of soccer players. Rehabilitation is another field where RPE is used to monitor training load (Noble and Robertson, 1996; Pena et al., 2003). This underlines the usefulness of RPE measures as indicators of strain, even in applied settings.

However, RPE is more than just an indicator of physical strain: according to the psychobiological model of endurance performance RPE limits how long people persist in an endurance task (Marcora, 2008, 2009; Marcora et al., 2008). This proposition is derived from motivational intensity theory (Brehm and Self, 1989; Richter et al., 2016) which constitutes the theoretical basis of the psychobiological model. The theory asserts that people adjust their effort to increasing task difficulty as long as the exertion of effort seems both justified and possible. The psychobiological model applies this reasoning to endurance performance, suggesting that people terminate a strenuous task either after having reached their justified level of effort or when further investment of effort seems impossible. Accordingly, exhaustion and task termination should not be determined by physiological indicators of strain, but rather reflect a deliberate decision to disengage from the task at hand when the justified or possible effort limit is reached (Pageaux, 2014).

This theoretical approach represents a marked shift from purely physiological models of (endurance) performance to psychologically grounded models. Empirical support for the propositions of the psychobiological model has accumulated in recent years (e.g., Marcora et al., 2009; Marcora and Staiano, 2010; Blanchfield et al., 2014a,b; but see also, Hureau et al., 2016). In a frequently cited study, participants performed a cycling task at 80% of their aerobic capacity until they were unable to produce the required power (Marcora and Staiano, 2010). Immediately after task termination, maximum voluntary cycling power (MVCP) of participants was measured. MVCP produced by participants was more than 300% of the power participants had to perform in the cycling task they just terminated. This supports the claim that physiological strain (e.g., muscle fatigue) is not the ultimate reason for task termination. However, a very strong association of RPE and task termination was observed, suggesting that RPE was the primary limiting factor of exercise termination.

The key property of the psychobiological model is its explicit acknowledgment of psychological factors that determine (endurance) performance (Pageaux, 2014). Indeed, it has been observed that psychological manipulations can have an effect on endurance performance via regulating RPE (Blanchfield et al., 2014a,b). Although research shows that endurance performance can be affected by subtle psychological manipulations that change RPE, the effectiveness of self-regulation strategies for down-regulating RPE and the corresponding effects on performance have not yet been documented.

In the present research, we turned to the self-regulation strategy of making *if-then* plans (so-called implementation intentions; Gollwitzer, 1999, 2014). It is plausible that recreational and professional athletes routinely make plans to regulate their effort and exertion (e.g., planning how to deal with feelings of pain or exhaustion during physical exercise). Furthermore, if-then planning in sport is regularly recommended by institutions, the public press (e.g., Calder, 2009; Gregoire, 2016), and by the scientific community (Achtziger et al., 2008; Brick et al., 2016; McCormick et al., 2016). However, to date it is not clear whether planning how to deal with effort can indeed help people down-regulate their RPE and thereby enhance endurance performance.

Implementation intentions are if-then plans in which people link critical situations and goal-directed behaviors: “*If I encounter Situation S, then I will perform Behavior B!*” This facilitates goal attainment beyond forming mere goal intentions (e.g., “*I want to achieve Outcome O/perform Behavior B!*”) across a variety of domains (e.g., health, academic, interpersonal; Gollwitzer and Sheeran, 2006; Adriaanse et al., 2011b; Belanger-Gravel et al., 2013; Hagger and Luszczynska, 2014). In the domain of endurance performance, for instance, if-then plans could specify how to deal with negative sensations during endurance performance (e.g., Thürmer et al., 2017). Forming implementation intentions promotes goal attainment because the situation specified in the if-part becomes mentally activated and receives attentional and perceptual priority (Wieber and Sassenberg, 2006; Achtziger et al., 2012; Janczyk et al., 2015), making it easy to detect and recognize (Aarts et al., 1999). Additionally, a strong associative link is established between the situation and the goal-directed behavior specified in the then-part that automates behavior (Gollwitzer and Brandstätter, 1997; Brandstätter et al., 2001; Bayer et al., 2009). With implementation intentions, people delegate their action control to situational cues (i.e., they create “instant habits;” Gollwitzer, 1999). This applies to external states as well as to internal, subjective states (e.g., feeling exhausted or stressed; Achtziger et al., 2008; Varley et al., 2011), under the premise that people can recognize these internal states when they occur. Thus, it seems plausible that people can form implementation intentions in which they plan how to deal with sensations of exertion (i.e., RPE) in an endurance task. The corresponding goal-directed behaviors (i.e., to ignore aversive sensations and keep going) should be automatically initiated when people get increasingly exerted.

In terms of the psychobiological model, implementation intentions could enhance endurance performance either by delaying the increase of RPE and/or by justifying higher levels of effort. Prior research has observed that people with implementation intentions perceive performance as less straining than participants with mere goal intentions and are therefore more likely to persist when facing increasing difficulty (Freydefont et al., 2016; Legrand et al., 2017). However, even after having formed implementation intentions people disengage from goals that become excessively difficult to attain, for instance when facing significant monetary losses (Legrand et al., 2017, Exp. 3). These findings suggest that implementation intentions can reduce perceived effort of performing goal-directed behaviors, however, without justifying additional effort (i.e., flexible tenacity; Gollwitzer et al., 2008).

Taken together, RPE appears to be a crucial limiting factor of performance and is a very influential construct in exercise physiology and psychology (Pageaux, 2016). We investigated whether the self-regulation strategy of forming implementation intentions enables people to down-regulate RPE and thus enhance their endurance performance. We expected a slower increase in RPE in the implementation intention condition compared to a control condition, without differences in the maximum RPE-value. Moreover, we hypothesized that this would translate into better performance in terms of time to failure and/or quality.

## Methods

### Participants and design

We recruited a sample of 62 female students (age: *M* = 23.7, *SD* = 4.0). We determined that this would allow us to detect differences between conditions of *d* = 0.65 at 95% power, a typical effect size when comparing implementation intentions to a control condition (meta-analysis by Gollwitzer and Sheeran, 2006). Our participants reported to engage in *M* = 3.4 (*SD* = 2.2) h of sport per week, 25.9% of which they ascribed to strength training. Four participants reported to be not actively engaged in any sport, while the remaining participants performed their main sport for *M* = 5.1 (*SD* = 5.4) years.

Only participants with no current or recent injuries of shoulders, arms, or the back were eligible for participation. Further, participants were instructed to refrain from consuming caffeine in the 2 h prior to the study. All participants complied to these restrictions. In a final questionnaire, we asked participants whether they had engaged in an exercise (10 participants in each conditions answered “yes”) or consumed alcohol the day before (Three participants in the control and two in the implementation intention conditions answered “yes”). The conditions did not differ with regard to these questions, *p*s > 0.78.

Upon their arrival at the lab, we randomly assigned participants to a control condition (*n* = 33) or an implementation intention condition (*n* = 29). Participants signed an informed consent that was approved by the Ethics Committee at the University of Konstanz (approval #24/2016). We compensated participants with 5 Euro or course credit.

### Measures and apparatus

#### Static muscular endurance task

**Figure 1:**
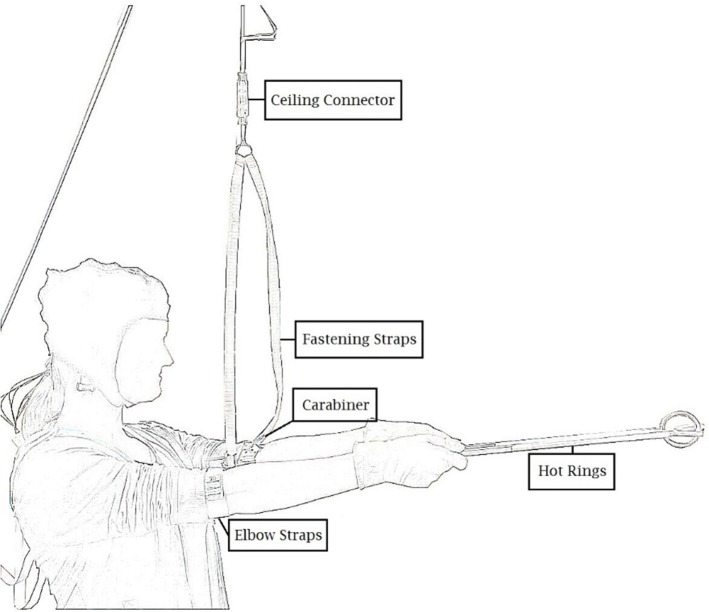
Schematic illustration of the static muscular endurance task.

In cooperation with the scientific engineering service at the University of Konstanz we developed a static muscular endurance task (introduced to participants as the “hot rings task”) that allowed us to reliably measure both the duration (i.e., time to failure) as well as the accuracy of performance. We instructed participants to hold two aluminum bars connected by two intertwined rings (length = 46.5 cm; weight of each bar = 55 g) for as long (duration) and with as few contacts between the rings (errors) as possible. Participants stood upright with their arms strapped into a holding device that was connected to the ceiling of the laboratory and comprised a connector element (see Figure 1). Prior to the task, the connector element was locked and the holding device was individually adjusted to participants' height so that their outstretched arms formed a 90° angle with their torso. The connector element was then unlocked at the beginning of the task, causing it to unplug and terminate the task as soon as participants' arms dropped below the preset 90° angle. To continuously track the accuracy of on task performance, a recording box was connected to the bars and registered ring contacts at 50 Hz.

#### Ratings of perceived exertion (RPE) and pain

Every 25 ± 10 s participants reported their perceived exertion (RPE) and pain using Category Ratio 10 (CR10) scales (Borg, 1998, 2004). People can distinguish RPE from other exercise-related sensations (Pageaux, 2016). Care needs to be taken to specify precisely what internal sensations participants should attend to (Pageaux, 2016). To ensure participants' differentiated perceived exertion from pain, we explicitly defined RPE as “the conscious sensation of how hard, heavy, and strenuous a physical task is” (Marcora, 2010). The two scales ranged from 0 (“nothing at all”) to 10 (“maximal”) or 11 (“even more than max”) (Pageaux, 2016). We printed them on separate sheets of paper placed on a wall in front of the participants.

### Procedure

Upon their arrival in the lab, participants were familiarized with the static muscular endurance task. They were instructed to hold the bars for as long as possible while avoiding contacts between the rings. We adjusted the holding device and informed participants about how to use the CR10 scales for rating their perceived exertion and pain (Pageaux, 2016). Participants then went through a brief demonstration in which they deliberately lowered their arms until the connector element unplugged.

After the demonstration was completed, we released participants from the apparatus and provided them with different instructions according to their condition. In the control condition, we instructed participants to repeat the task instructions (i.e., “*The task is to persist for as long as possible while avoiding contacts between the rings!*”). In the implementation intention condition, we instructed participants to phrase the instructions in terms of a goal intention (i.e., “*I want to persist for as long as possible while avoiding contacts between the rings!*”) and to furnish it with an if-then plan: “*If the task becomes too strenuous for me, then I ignore the strain and tell myself: Keep going!*”

Finally, participants were again strapped into the apparatus and raised their arms into the 90° position. To start the task, we then unlocked the connector element, switched on the recording box, and started a timer to measure time to failure. While participants engaged in the task, they received no verbal feedback and the experimenter remained outside their field of vision to minimize experimenter influence. For the same reason, the verbal prompts for the CR10 scale ratings (i.e., the words “effort” and “pain”) were generated using “Text 2 Speech Pro” (Mattos, 2016) and played by the computer. Answers were documented by the experimenters. The same two female experimenters collected all data for this study.

The task was terminated as soon as the connector element unplugged and participants were released from the apparatus. The experiment concluded with a final questionnaire comprising six items to measure how strongly participants were committed to perform well in the task (α = 0.78; e.g., “It was important for me to persist for as long as possible in the endurance task,” “I did not care how precisely I worked on the endurance task [reversed]”) on 7-point Likert scales (1 = *does not apply*, 7 = *fully applies*) and demographic questions (e.g., age, gender, physical activity).

## Results

### Data analysis

We report independent *t*-tests comparing the two conditions and applied Welch-corrections when the assumption of homogenous variances was violated. We augment this by also reporting non-parametric Wilcoxon rank sum tests because values were often not normally distributed. As all dependent variables except time to failure were measured repeatedly during the task, we submitted them to models with Condition (control vs. implementation intention) as between-participants and Time-to-Failure ([0–10%] vs. (10–20%] vs. …vs. (90–100%]) as within-participants factor. There were, however, empty cells in the design resulting from early quitters, preventing us from using standard mixed ANOVAs. Instead, we turned to mixed-effects models for which empty cells are no issue and do not require removing observations from the dataset.

All analyses were run in the statistical software environment R (3.3.1, R Core Team, 2016). We estimated mixed-effects models with lme4 (1.1–12; Bates et al., 2015) and used the Satterthwaite approximation for degrees of freedom implemented in lmerTest (2.0- 32; Kuznetsova et al., 2016) to establish the significance of fixed-effects. Effect sizes were computed with the effsize package (0.6.4; Torchiano, 2016) and plots were created using ggplot2 (2.2.1, Wickham, 2009).

### Task commitment

Participants in the implementation intention condition (*M* = 6.0, *SD* = 0.7) reported lower task commitment than participants in the control condition (*M* = 6.3, *SD* = 0.6) after having completed the task, and this difference approached significance, *t*_(60)_ = 1.87, *p* = 0.066, *g* = 0.34, and *W* = 602.5, *p* = 0.079.

### Task performance

#### Time to failure

**Figure 2:**
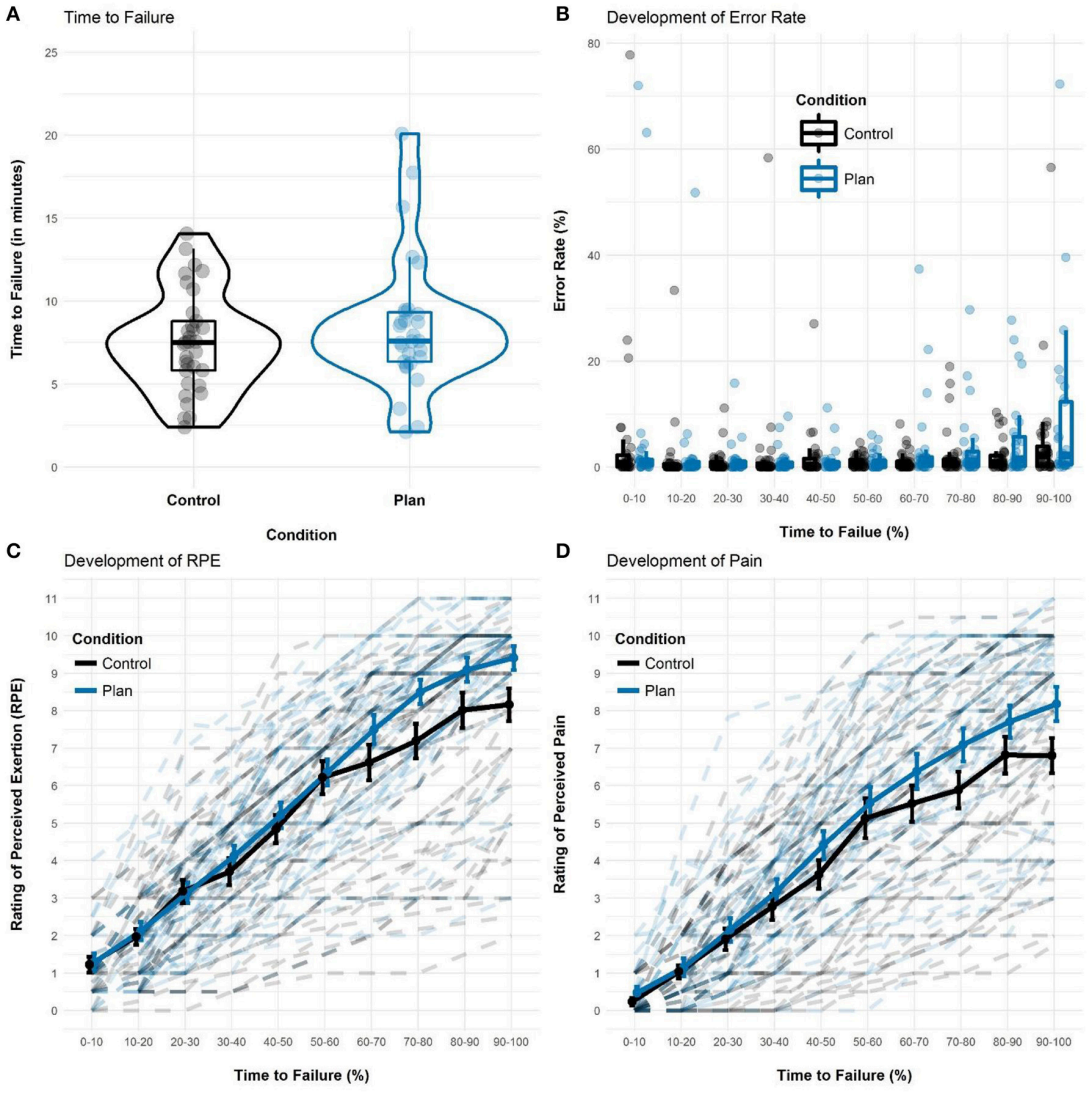
Visualization of results as a function of Condition and Time-to-Failure. Groups did not differ in how long **(A)** and how accurate **(B)** they persisted in the static muscular endurance task. Significant between group differences and Time-to-Failure × Condition interactions emerged for RPE **(C)** and in attenuated form for perceived pain **(D)** as well. Error bars in **(C)** and **(D)** represent standard errors of the mean.

Descriptively, participants in the implementation intention condition (*M* = 8.5 min, *SD* = 4.0) performed the endurance task about 1 min longer than control participants (*M* = 7.5 min, *SD* = 3.0), but this difference was not significant, *t*_(60)_ = 1.10, *p* = 0.274 and *W* = 408, *p* = 0.326 (Figure 2A).

#### Error rates

We found a significant effect of Time-to-Failure on error rates, *F*_(9, 540)_ = 4.69, *p* < 0.001, reflecting that participants committed more errors as the task progressed. Neither the main effect of Condition, *F*_(1, 60)_ = 0.80, *p* = 0.374, nor the interaction effect of Condition and Time-to-Failure, *F*_(9, 540)_ = 0.93, *p* = 0.494, reached significance, indicating that goal and implementation intention participants did not differ in their susceptibility to committing errors (Figure 2B).

### Effort and pain ratings

#### Ratings of perceived exertion (RPE)

We found a significant main effect of Time-to-Failure, *F*_(9, 462.94)_ = 470.66, *p* < 0.001, indicating that RPE increased over the endurance task. We also observed a significant interaction of Condition and Time-to-Failure, *F*_(9, 462.94)_ = 3.77, *p* < 0.001. As illustrated in Figure 2C, this interaction effect reflects a steeper increase in RPE among implementation intention participants after exceeding a rating of 6–7 on the CR-10 scale. In the final 10% of the task, implementation intention participants (*M* = 9.4, *SD* = 1.7) reported significantly higher RPE than control participants (*M* = 8.2, *SD* = 2.5), *t*_(56.8)_ = 2.09, *p* = 0.026, *g* = 0.41 and *W* = 318, *p* = 0.020.

#### Ratings of perceived pain

We found a significant main effect of Time-to-Failure, *F*_(9, 463.29)_ = 296.19, *p* < 0.001, indicating that perceptions of pain increased over the endurance task. The interaction effect of Condition and Time-to-Failure approached significance as well, *F*_(9, 463.29)_ = 1.81, *p* = 0.064. As illustrated in Figure 2D, this pattern of results reflects a steeper increase in perceived pain among implementation intention participants after exceeding a rating of 5–6 on the scale. In the final 10% of the task, implementation intention participants (*M* = 8.2, *SD* = 2.5) reported significantly more intense pain than control participants (*M* = 6.8, *SD* = 2.7), *t*_(60)_ = 2.09, *p* = 0.041, *g* = 0.38 and *W* = 326.5, *p* = 0.031.

## Discussion

In this study, we showed that the self-regulation strategy of forming implementation intentions affects ratings of perceived exertion (RPE) during a static muscular endurance task. Unexpectedly, implementation intention participants who planned to ignore sensations of strain reached higher RPE values before terminating the task while experiencing a significantly faster increase of RPE than control participants. Time to failure and quality of performance was similar between conditions. It thus seems that forming implementation intentions raised the level of effort participants were willing or able to invest but at the same time also increased the rate at which RPE accrued over time. As RPE is among the most frequently assessed concepts in exercise physiology and implementation intentions are a widely recommended and used self- regulation strategy these findings are very important for at least two reasons.

### Plans affect RPE

First, this study underlines how readily RPE (which is now widely accepted as a central correlate of physical strain) can be altered by a psychological manipulation. This is important because there is still an ongoing debate regarding *which* sensory signals contribute to what extent to the perception of effort (Pageaux, 2016). From a *peripheral* point of view, RPE is a function of afferent signals that originate from peripheral organs involved in a specific task (i.e., high correlations of RPE with heart rate and other physical indicators of strain are consistent with this interpretation). In a particularly convincing study, Amann et al. (2010) showed that partially blocking sensory afferents (by lumbar intrathecal injection of fentanyl) from working muscles led to reduced RPE in a cycling task. Thus, from a peripheral perspective, reduced somatosensory feedback results in a lower perception of effort.

From a *central* point of view, perception of effort is independent from such afferent feedback (Marcora, 2009). To support this claim, Marcora et al. (2008) experimentally dissociated muscle fatigue from the metabolic stress associated with the fatiguing task (metabolic stress in turn stimulated afferent signals) and showed that locomotor muscle fatigue alone led to higher RPE. Thus, maintaining performance when fatigued is associated with increases in central motor command and this is in turn is perceived as effortful. Consequently, from a central point of view, perception of effort is the result of a “conscious awareness of the central motor commands to the locomotor and respiratory muscles (Marcora, 2009, p. 2061).” Our experimental design was not set out to test peripheral and central explanations of RPE against each other. However, our results fit very well into the central explanation: Psychological manipulations can affect attendance to internal and external states (e.g., Pennebaker and Lightner, 1980) and the psychobiological model of endurance performance proposes that psychological manipulations can affect RPE. In our case, participants in the implementation intentions groups were possibly more aware of their sensations while performing the task. This might have caused the increase in perceived exertion. In line with the idea that implementation intentions led to a greater awareness, participants in this group also reported to perceive more pain.

### Ironic effects of plans on RPE

Second, our results show that the effects of psychological manipulations on RPE and performance are not necessarily straightforward. Contrary to our predictions, implementation intentions participants did not manage to persist longer in the muscular endurance task than goal intention participants. First, they reached higher RPE scores than control participants before disengaging from the task, which could be interpreted as an increased justification of effort. However, this is at odds with the observation that implementation intention participants tended to be less committed to perform well than control participants; moreover, prior research suggests that implementation intentions do not justify additional effort. A cautious explanation might be that participants effectively implemented the “keep going” part of their plan. From this perspective, the higher final RPE scores might be a volitional rather than a motivational effect. Second, RPE rapidly increased among implementation intention compared to control participants, with a steep slope after having passed about the middle of the scale. To illustrate, RPE values in the implementation intention condition exceed those in the control condition by about 1.5 points after 3/4 of the time to failure. Both effects—the higher overall RPE before task termination and the steeper increase—seem to have canceled out each other, rendering time to failure similar between conditions. We did not expect this pattern of results; however, we would like to offer a tentative explanation for our finding.

The implementation intention instruction prompted participants to ignore strong sensations of exertion. Contrary to what one would expect under such an instruction, however, implementation intention participants experienced even stronger sensations of exertion than control participants once they had exceeded the middle of the RPE scale. This observation fits well into ironic processing theory (Wegner, 1994), which asserts that mental control is based on two interacting processes: (1) an intentional and effortful operating process that creates thoughts and sensations consistent with a desired state and (2) an automatic and non-conscious monitoring process that searches for mental contents indicating control failure. As long as mental capacity is sufficient, the operating process can successfully ignore or suppress unwanted thoughts and sensations. When mental capacity becomes scarce (e.g., due to physical strain, stress, or cognitive load), however, the operating process is derailed more strongly than the monitoring process, which continues to search for contents indicating failed mental control. As a consequence, the very thoughts or sensations that one strives to ignore or suppress are amplified rather than attenuated. In terms of ironic processing theory, implementation intention participants might thus have suffered more strongly from intruding sensations of exertion once their mental capacities were limited by the straining task performance.

Consistent with this interpretation, ironic effects of forming implementation intentions have been observed in other domains as well, especially when the plan negated the execution of a behavior (e.g., planning what not to eat; Adriaanse et al., 2011a). Yet, this does not imply that implementation intentions necessarily suffer from ironic effects. First, there are also studies showing that even negation implementation intentions can be effective (e.g., Verhoeven et al., 2017). Second, implementation intentions specifying to ignore undesired critical cues like intrusive thoughts and negative affect also work well (e.g., Achtziger et al., 2008; Gallo et al., 2009). Finally, a study using negation implementation intentions to regulate perceived pain during an endurance task (Thürmer et al., 2017) has successfully enhanced group performance, however without explicitly measuring perceived pain. At the bottom line, ironic processing theory provides both a compelling and a parsimonious explanation of our present results and implementation intentions seem to be susceptible to ironic effects, even though the exact circumstances of their occurrence have yet to be explored.

## Conclusion

In the present research, we set out to explore whether people can use the self-regulation strategy of making if-then plans (implementation intentions) to down-regulate RPE and thereby enhance their endurance performance. While our results demonstrate that planning to ignore sensations of strain indeed affects RPE, it did so in an unexpected and ironic way—increasing both the rate at which RPE accrued and the absolute RPE limits that people achieved before terminating the task. This observation is important given how frequently RPE is used as a correlate of physiological processes that ultimately limits performance. Moreover, the finding that implementation intentions can backfire in sport settings is of great importance as well because planning how to deal with thoughts and sensations during physical performance is presumably widespread among recreational and professional athletes, whether these plans are made consciously or not. Making plans is also recommended by the scientific community (e.g., Achtziger et al., 2008; Brick et al., 2016; McCormick et al., 2016) and part of psychological skills trainings that aim to improve sports performance (e.g., Birrer and Morgan, 2010). Our results show that athletes and coaches who use plans to ignore aversive sensations of exertion might in turn suffer from even more severe sensations of exertion, which might easily impair performance. As such, the present research contributes to the existing literature on ironic processes in sport (Janelle, 1999).

## Ethics statement

This study was carried out in accordance with the recommendations of the ethics guidelines of the University of Konstanz' ethics committee with written informed consent from all subjects. All subjects gave written informed consent in accordance with the Declaration of Helsinki. The protocol was approved by the ethics committee of the University of Konstanz (approval #24/2016).

## Author contributions

MB and WW developed the research question and conducted the empirical part of the study. MB conducted the statistical analyses. MB and WW then analyzed the results, and jointly wrote the manuscript.

### Conflict of interest statement

The authors declare that the research was conducted in the absence of any commercial or financial relationships that could be construed as a potential conflict of interest.
